# Context Management Platform for Tourism Applications

**DOI:** 10.3390/s130708060

**Published:** 2013-06-24

**Authors:** David Buján, David Martín, Ortzi Torices, Diego López-de-Ipiña, Carlos Lamsfus, Joseba Abaitua, Aurkene Alzua-Sorzabal

**Affiliations:** 1 Deusto Institute of Technology, DeustoTech, University of Deusto, Avda. Universidades 24, 48007 Bilbao, Spain; E-Mails: dipina@deusto.es (D.L.-I.); joseba.abaitua@deusto.es (J.A.); 2 Cooperative Research Centre in Tourism, CICtourGUNE, Mikeletegi Pasealekua 71, Donostia-San Sebastián 20009, Spain; E-Mails: DavidMartin@tourgune.org (D.M.); OrtziTorices@tourgune.org (O.T.); CarlosLamsfus@tourgune.org (C.L.); AurkeneAlzua@tourgune.org (A.A.-S.)

**Keywords:** context modelling, knowledge representation and management, ontologies, Open Data, Linked Data, tourism

## Abstract

The notion of context has been widely studied and there are several authors that have proposed different definitions of context. However, context has not been widely studied in the framework of human mobility and the notion of context has been imported directly from other computing fields without specifically addressing the tourism domain requirements. In order to store and manage context information a context data model and a context management platform are needed. Ontologies have been widely used in context modelling, but many of them are designed to be applied in general ubiquitous computing environments, do not contain specific concepts related to the tourism domain or some approaches do not contain enough concepts to represent context information related to the visitor on the move. That is why we propose a new approach to provide a better solution to model context data in tourism environments, adding more value to our solution reusing data about tourist resources from an Open Data repository and publishing it as Linked Data. We also propose the architecture for a context information management platform based on this context data model.

## 1. Introduction

The customization of information in mobile scenarios is quite crucial since the user is on the move and he/she usually requires a very specific information at a given time and place. That is especially the case of the tourism domain, where visitors need personalised information about points of interest and nearby activities. This way, it is crucial for services to be able to acquire data about the visitor's context in order to adapt the functionality of the system to the gathered data, but this personalization process is not a trivial task, because data from different context sources has to be acquired and processed by context-aware systems in order to provide the user with the needed information. This processing involves the population of a context data model and its management in order to adapt the system's behavior to the situation of the visitor.

Context has been widely studied and there are several authors that have proposed different definitions. A first group of authors consider context as the surroundings of the interaction between the user and the application [1–3]. A second group of authors consider the tasks of the user as the main context information for the system [4,5]. A third group of authors consider that context is the required information to characterize the situation of an entity [6–8].

In mobile environments, such as the tourism domain, location is the main context information to be considered in order to adapt the behaviour of a context-aware tourism system [9]. Most of the existing commercial mobile guides [10] show that most of the mobile tourist services either provide location-based information or concentrate on delivering personalized information. One of the major drawbacks of all these systems is the amount of (active) interaction they require from the user point of view. These are examples of a combination of both [11–13]. However, context has not been widely studied in the framework of human mobility and the notion of context has been directly imported from other computing fields without specifically addressing the tourism domain requirements, and in particular the visitor's needs.

Context has traditionally been studied in other research disciplines [14]. Most of the existing context data models are designed to be applied in general ubiquitous computing environments and do not contain specific concepts related to the tourism domain. There are some approaches to context modelling in tourism but they do not contain enough concepts to represent context information related to a visitor on the move. In this article, we propose a new approach to provide a better solution to context data modelling in tourism environments. This solution adds value reusing Open Data about tourist resources from the Open Data Euskadi initiative and publishing it as Linked Data. A tourism information system, called TourExp, has been implemented in order to validate this new approach. This system includes a content management system where tourist providers (owners of tourist resources or travel agencies) can publish their information and tourist offer (activities and packages) and the staff of the Basque Tourism Agency (tourist technicians and area managers) can organize this touristic offer in terms of travel experiences using several criteria. It also provides mobile applications for visitors on the move to consume this information and get suggestions provided by a recommendation system based on visitors' context (out of the scope of this paper). The research work presented in this paper refers to the context management platform developed in the project as a framework for different subsystems and applications acting as context information providers and consumers.

The rest of this paper is structured as follows: in Section 2, we introduce our context data model, remarking aspects to consider for context management and some particular details of our solution, including how the information represented by our data model is published. Section 3 describes the architecture designed for the TourExp context management platform and its implementation. Finally, we describe our work in progress in Section 4, referring to different applications that are being developed in this project and will access TourExp context information.

## 2. Background

In order to store and manage context information a context data model is needed. There are several data models that can be used in order to represent context information [15]. These are classified by the data structures that are used to store contextual information:

- *Key-value pairs*: this is the most simple data structure for modelling contextual information. This data model is easy to manage, but lacks capabilities for enabling efficient context retrieval algorithms and data inference mechanisms.
- *Markup scheme models*: this model is based on a hierarchical data structure consisting of tags with attributes and content. An example of this approach is the extensible markup language (XML).
- *Object oriented models*: this approach is based on the benefits of encapsulation and reusability. An example of these kinds of modelling techniques is the Java programming language, which is based on classes and objects to represent data.
- *Ontology Based Models*: ontologies can specify concepts with properties and inter-relations between those concepts. Also, they offer a very expressive language in order to define axioms and restrictions over those concepts.

An exhaustive analysis of context management methods [15] indicates that ontologies are the most adequate tool for context information management. Thus, several authors working on context-awareness converged upon a fact: context information and context models could well be handled using semantic technologies. This would set the way for systems to more easily share and re-use context and moreover, it would support to not only check the model's consistency, but also to infer implicit context knowledge by the reasoning capabilities of ontologies [16]. One of the first examples of context ontologies is the Context Broker Architecture (CoBrA) ontology [17]. It is expressed in OWL and it represents a collection of terms describing places, software agents, events, and their associated properties.

The Standard Ontology for Ubiquitous and Pervasive Computing (SOUPA) ontology is an evolution of the previous ontology [18]. It represents a shared ontology that includes modular vocabularies to represent software agents with associated beliefs, desires and intentions, time, space, events, user profiles, actions and policies for security and privacy. It is divided in two different ontologies, namely SOUPA-Core and SOUPA-Extensions. SOUPA-Core defines a set of classes and entities common to almost all scenarios within pervasive computing, while SOUPA-Extension ontologies extend from the Core and define additional vocabularies to support specific domains.

The GAS ontology [19] was developed in order to semantically describe the basic concepts within a ubiquitous computing environment. Its main objective was to provide a common vocabulary for heterogeneous devices that constitute a pervasive computing environment.

The ONCOR [20] ontology is basically thought to provide a flexible and practical ontology to describe locations, devices and sensors within ubiquitous computing systems that delivers personalized information in a building environment.

On the one hand, all the above ontologies are designed to be applied in general ubiquitous computing environments, but do not contain specific concepts related to the tourism domain. On the other hand, there are several ontologies in order to represent the tourism domain [21–24], but these approaches do not contain concepts to represent context information related to the visitor. This way, there is a need to extend and merge the existing modelling approaches in order to provide a better solution to model context data in tourism environments.

## 3. TourExp Context Model

The main goal of this research work is to define the steps to follow when analyzing mobility users, *i.e.*, users of mobile technologies that are constantly on the move. There is also a definition of such users' context so that offers made by the system are bound to valid criteria. As explained in the introduction, visitors are mobility users that have needs of specific information on the go such as information about events, information about interesting places to visit and so on. Context-aware systems are ideal to cover those needs because they take into account data about the user (visitor) like location, social context, preferences, device types, weather conditions, *etc.*

### 3.1. Aspects to Consider for the Context Management

It was decided to take into account two kinds of aspects when managing context information: human factors (those of the own user) and environmental factors (those that have to do with the environment).

#### 3.1.1. Human Factors

##### Information about the user

User's identifier (e-mail), password, gender, country of origin, birth date, physical limitations (blindness, reduced mobility, deafness, *etc*), feeding intolerances, religious tendencies and preferences.

##### Social environment of the user

Information related to the user's social networks (Facebook, Twitter, FourSquare, *etc.*) and travel type (family/ friends/ business/ couple).

#### 3.1.2. Environmental Factors

##### User location history

The tracking of the travelers can be made by GPS or by detecting the placement of the network they are using at the moment. Apart from the actual localization, TourExp context model allows to represent historical data about the traveler's placement.

##### Infrastructure

Having information about public transports and other tourist resources is useful for travelers on the move. Because of this, the recommendation module can grant such information to the user describing the list of resources close to him/her at the time or describing the resources related to the experience selected.

##### Weather

The weather forecast is retrieved from web services like Yahoo Weather so the recommendation module could suggest suitable activities in each case.

#### 3.1.3. System Factors

##### User's tasks

Track of the user's interactivity with the system to register information about bookings, ratings, selected favourites, check-ins, *etc.* In addition to this, it is relevant to save information about the travels made by the user and keep them saved on the travel profile (type and motivation of the travel, cost, duration, area of activity, *etc*). This way, it is possible to make suggestions to the user and create user profiles from the recommendation module that is being developed in this project.

##### User's access mode

The information given might vary depending on the device used by the traveler (mobile device or tourism platform “fixed access point”) and its technical features.

### 3.2. Data Modelling for Context Management

**Figure 1.**
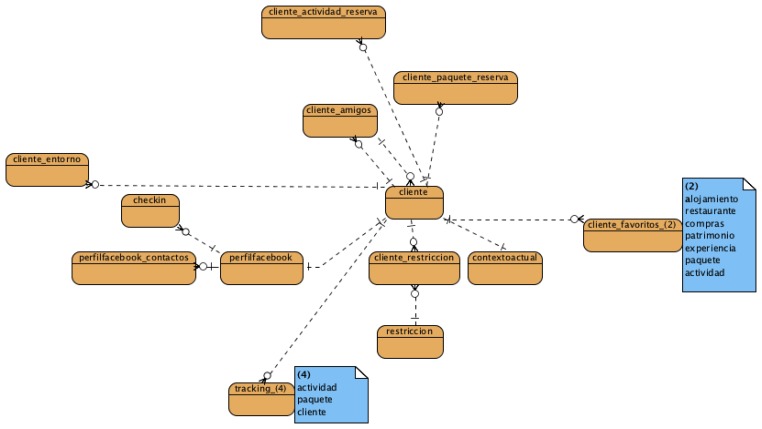
Diagram with some entities related to TourExp context data model.

The general data model that represents all the information related to TourExp system has been designed after the analysis made taking into account different input sources: tourist experiences defined at Euskadi Turismo website [25], open datasets at Open Data Euskadi initiative website [26] and requirements extracted from TourExp's applications. Figure 1 only shows part of the huge data model diagram related to context modelling entities related to some aspects considered in previous section and which are explained in detail in Section 4.2.1, where these tables are referred both in English and Spanish, the language used to present this research work and the one used for the implementation, available only in Spanish. These entities are related to others in the global TourExp data model whose development has involved some end users of the TourExp system like the Basque Tourism Agency and some tourist providers in order to validate the design decisions.

Besides, there are some other aspects of the user context modelling that affect the development of certain modules of the project and that are represented in our model, such as TourExp does not keep track of the use made of it by not logged in users, locates the traveler periodically to keep a tracking record of him/her, relates the actual context of a user during a booking or stores ratings (experiences, activities, tourist resources) made by users.

Another remarkable aspect of TourExp developments is that retrieving personal data from a user's social network profile to fill the registration form makes it more user-friendly. On a side note, the data mining process being developed in this project is leading to create traveler profiles for describing the behaviour of the users and enabling the search of “twin-souls” within it to be used in the recommendation module.

Other approaches do not contain enough concepts to represent context information related to the visitor or tourist on the move as we need in the TourExp project, so we have proposed a new approach to model context data in tourism environments.

### 3.3. TourExp Vocabularies to Model Context Data in Tourism Environments

As explained before, the ontologies from related work designed to the tourism domain [21–24] and others like QALL-ME, Harmonise, Hi-Touch, DERI eTourism, cDOTT, Cruzar, ebSemantics and ContOlogy [27]. The list of vocabularies linked from TourExp Data Model is shown in Table 1, as well as some examples of mappings for a tourist resource (Table 2) and context information (Table 3).

Therefore, we have developed the TourExp ontology [28] for tourist resources and related concepts of the project and used ContOlogy [29] to represent as much context information as possible. Some other entities and properties that are not represented in these ontologies have been linked to standard vocabularies from the Linked Open Data cloud like the WGS84 geo-positioning W3C vocabulary or the DBpedia ontology. Since the TourExp data model is very huge, most of the entities and properties are mapped to a TourExp vocabulary named “vocab”.

## 4. TourExp Context Management Platform

The previous context data model can be by other modules of the TourExp system, which general architecture is described in detail in the corresponding deliverables of the project, but this is out of scope of this paper. In this article we will focus on the context management subsystem, which involves three components from the global TourExp system:

- *Context manager*. This component, described in next subsection, is in charge of gathering, modelling, storing, inferring and querying context information using a knowledge base.
- *Context consumers*. These applications use context information to provide their functionality: recommendation services, content management services, *etc.*
- *Context providers*. This set of devices, services or applications provide real data to populate the context data model.

This context management subsystem is connected to the rest of the TourExp system, where every application can play both consumer and provider roles in different moments. Next section describes the architecture designed for our TourExp context management platform and its implementation.

### 4.1. Platform Architecture

**Figure 2.**
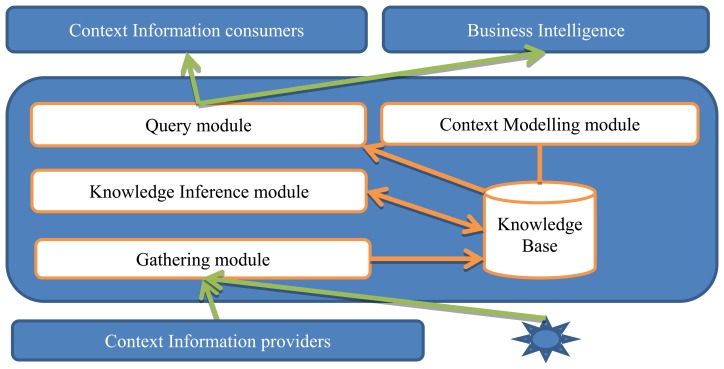
Architecture of the TourExp context management platform.

The context manager is composed by five modules (as shown in Figure 2) that are in charge of gathering, modelling, storing, inferring and querying context information using a knowledge base:

The *Context Modelling module* is in charge of identifying and determining the set of attributes that will be represented in the TourExp context data model for a customer/visitor. This context data model is integrated with the TourExp global data model and includes information about the customer's profile, his/her Facebook profile, travel profile, customer's locations, weather conditions and tourist resources selected as favourites by a customer, as described in Section 3.

The *Knowledge Base* component is implemented as a set of tables representing the context data model that are created and related to the rest of tables in the TourExp global database, as well as a dynamic provider of context information in RDF format. The context information providers store their data in these tables and context information consumers can query this data by means of two access points described in the Query module.

The *Gathering module* is in charge of storing context information from different internal or external sources or providers identified in TourExp, like devices used by a customer/visitor to access the TourExp applications (smartphones, tablets, built-in access points) and external web services. A set of web services receives this context data to be processed and stored in the Knowledge Base, in order to be consumed by other modules or TourExp applications later.

The *Query module* provides two different access points to the context information stored in the Knowledge Base. This module provides interfaces for context information consumers like the TourExp recommendation system, the Business Intelligence system or the global TourExp Content Management system. These context information consumers can invoke a set of web services to get context information in JSON and XML format or query a SPARQL endpoint to get it in RDF format.

The *Knowledge Inference module* can obtain and deduce new information from the context data stored by the Gathering module related to a customer/visitor, like filling gaps in his/her customer's profile from his/her Facebook profile, detecting new relations among customers' profiles, travel profiles or experiences. This new information can be useful for context information consumers like the recommendation system during the process of selecting accurate tourist activities or resources for a customer/visitor depending on his/her context.

### 4.2. Platform Implementation

This section shows implementation details of each module of the architecture described before about the TourExp context management platform. The context information data model is implemented using a PostgreSQL database and the functionality to store and query context information is provided through RESTful web services deployed in a Tomcat server that use both JSON and XML formats for sending messages between the client and the server. A D2R server [30] is used to transform dynamically the content of the database in RDF format on demand and to provide a SPARQL endpoint to query this content using the SPARQL query language.

#### 4.2.1. Context Modelling Module

Several data have been considered as context information in TourExp. As described in Section 3, some human, environmental and system factors have been taken into account to develop the database model that stores the aforementioned context data. These data include information about the customer's profile, his/her restrictions or items selected as favourites, information about the customer's physical environment, weather conditions or means of transportation.

More precisely, human factors like user's details and his/her social environment are represented by these tables:

- *Customer* (*cliente*): this table stores information about the gender of the customer (visitor), the birthday date, the language, the country, the province and other typical fields used during the registration process at any application.
- *Costumer's restrictions* (*cliente restriccion*): this table stores the restrictions or disabilities provided by the customer during the registration process.
- *Customer's environment* (*cliente entorno*): this table stores the preferred kind of experiences for a certain customer.
- *Customer's Facebook profile* (*perfilfacebook*): the information gathered from Facebook Graph API with the explicit consent of the user is stored here and complement his/her profile with data like current state, preferred music, films, sports and likes on posts and photos. These data are obtained to be used in research. The use of these data for a commercial application would imply to sign a commercial exploitation contract.
- *Customer's Facebook contacts* (*perfilfacebook contactos*): this table stores the Facebook contacts of a certain user that are also registered in the TourExp platform.

The environmental factors (and some system factors related to access mode) are integrated mainly in this table, although there are other tables to store historical data:

- *Customer's current context* (*contextoactual*): data about the current context of the visitor are stored here. These data include information about the travel motivation (*i.e.*, business), travel mates (*i.e.*, family, friends), weather conditions, means of transportation (*i.e.*, train, bus), device type (*i.e.*, smartphone, tablet), GPS availability, number of travel days, accommodation type, current and previous location.

And finally, the system factors like the user's tasks (interaction of the user with TourExp and also Facebook) are implemented by these other tables (except the access mode):

- *Costumer's favourite items* (*cliente_favoritos* *): there is a table for each of the items and points of interests (experiences, activities, activity packages, restaurants, accommodations, heritage and shopping areas) considered in TourExp. This table stores a punctuation of the user between 1 and 5.
- *Customer's check-in actions* (*checkin*). This table stores the check-in actions carried out by the TourExp users using Facebook.

There is also a table to represent customer's tracking information (trackingcliente) and for storing historical context data of the customer. These data are processed by the knowledge inference module to classify customers in different travelers' profiles (as explained later in Section 4.2.5.). Besides, they are also exploited by the recommendation system in order to perform better suggestions to the users of TourExp system, which can be done using all or part of the factors mentioned before: his/her personal, social, environmental and/or system context, depending on the filters selected on the mobile application.

**Figure 3.**
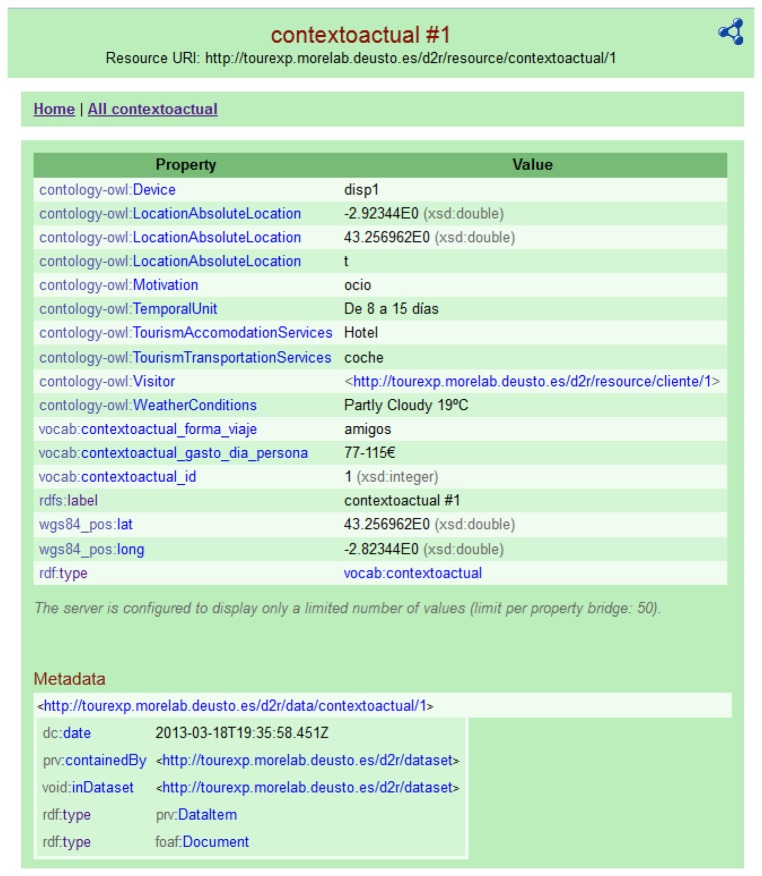
Example of current context information about a customer/visitor.

Figure 3 shows an example of current context information about a customer/visitor on the move.

#### 4.2.2. Knowledge Base

This component is implemented as a set of tables using a PostgreSQL database mentioned in the previous section, as well as a dynamic provider of RDF triples using a D2R server. This tool publishes the content of the database in RDF as Linked Open Data using the vocabularies described in Section 3 and enables RDF and HTML browsers to navigate the content of the database.

**Figure 4.**
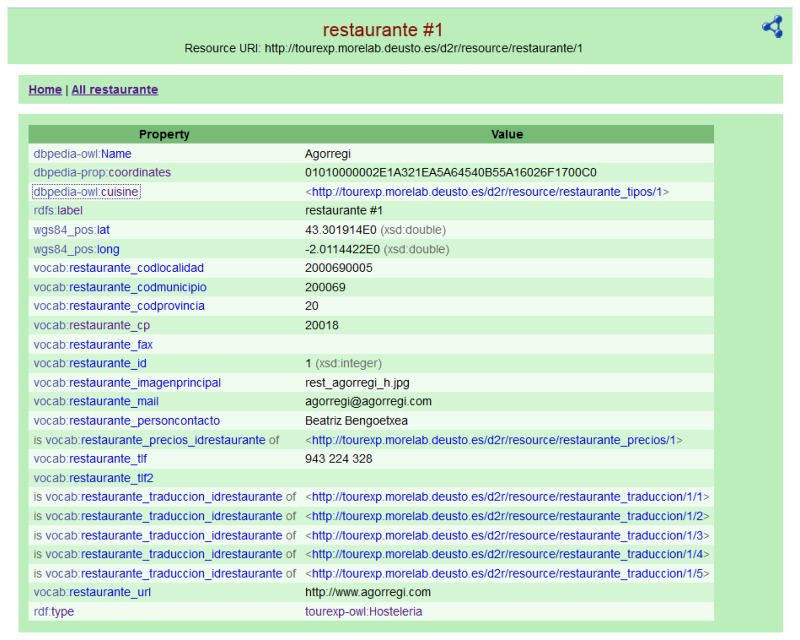
Example of navigable information in RDF format about a tourist resource.

D2R generates a mapping file that relates entities and columns from the database to entities and properties of ontologies and other well-known vocabularies. These mappings are used by D2R to generate RDF files dynamically or on-demand. We have ruled out a static RDF storage due to the dynamic nature of our database. Figure 4 shows an example of navigable information in RDF format about one row from a table available on the visual interface for any standard web-browser provided by our TourExp D2R instance [31].

#### 4.2.3. Gathering Module

The following section describes the available RESTful web services [32] for storing context data (shown in Table 4). These services have been grouped taking into account the elements of the TourExp context data model that are in charge of representing and storing the customer's location, current weather conditions, customer's profile, his/her current travel profile and items selected as favourites:

##### Customer's location

This service is in charge of storing current customer's location (latitude and longitude). This information is stored in the “contextoactual” table (CurrentContext). Besides, historical data about past locations is stored in the “tracking_cliente” table (customerTracking).

##### Current weather conditions

This service requests Yahoo Weather web service according to the gathered latitude and longitude of the user and gets the temperature data. Then, the “contextoactual” table (CurrentContext) is updated, but historical values of the weather are not stored.

##### Customer's profile

This service is used to store characteristic information related to a customer into “cliente” table (customer). The unique identifier for each customer is generated automatically. The typeOfProfile column is not stored here since it will be updated by the knowledge inference module establishing a relation between the customer's profile and traveler profiles, as explained in Section 4.2.5.

##### Customer's travel profile

This service stores information introduced by a costumer/visitor on the TourExp mobile application to describe the type of travel he/she is doing in terms of travel motivation (*i.e.*, business), the travel mates (*i.e.*, family, friends), *etc.* This information is stored in the “contextoactual” table (CurrentContext) and also saved periodically as mentioned before. These data will be used later by the TourExp system to infer types of travel profiles (defined by budget, travel motivation, travel mates, *etc.*). Moreover, the knowledge inference module aforementioned will use these data to establish relations between traveler profiles and types of travel profiles.

##### Customer's favourite items

This service stores information about the user's interaction with the TourExp application, regarding to items selected as favourites: type of experiences, activities, packages of activities and tourist resources like accommodations, restaurants, cultural heritage and shopping areas. All these data are stored in the “cliente_favoritos_*” tables (customer_favourites_*), where * is each of the mentioned items. These items can be valued from 1 to 5.

#### 4.2.4. Query Module

This component provides two different access points to the context information stored in the Knowledge Base:

- RESTful web services [33] (shown in Table 5) that return context information in JSON and XML format using this URLs for GET requests:
  ○ [URLBase]/clientes.xml
  ○ [URLBase]/clientes.json
- SPARQL endpoint [34] to get it in RDF format.

Most of these services have optional parameters like “start” (initial results of pagination: default = 0), “size” (page size: default = 10) and “language” (preference for results) and the search service has also these others: name, last Name, mail and phone Number.

**Figure 5.**
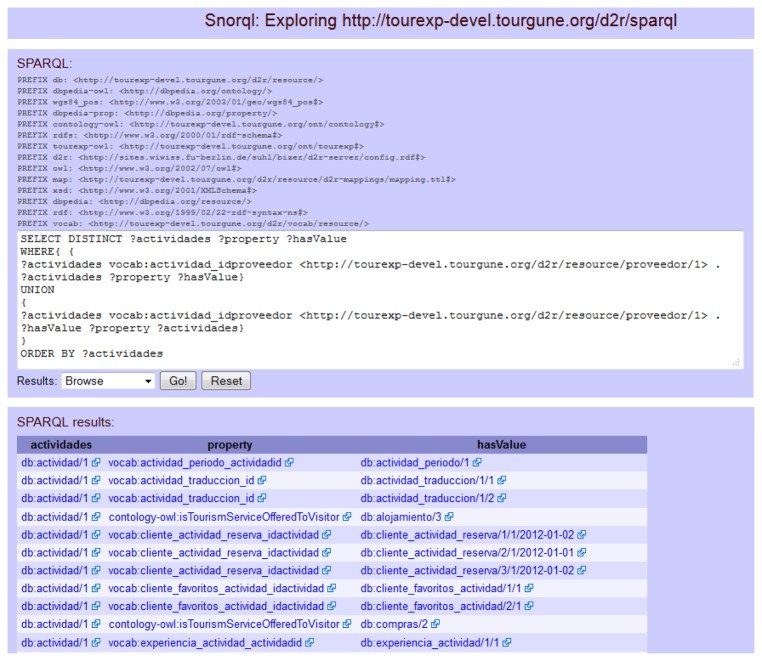
TourExp SPARQL visual browser and a query example.

The Query module also allows querying the database using the SPARQL query language to get RDF files dynamically by means of the SPARQL endpoint provided by the TourExp D2R server instance. This is not only another way of accessing context data, but also a more powerful tool that allows complex queries to get implicit knowledge that is not made explicit using a traditional relational database. This can be achieved thanks to the established semantic relations among terms of the different ontologies and vocabularies used to map tables and columns from the TourExp database to entities and properties of the mentioned ontologies and vocabularies. Figure 5 shows the SPARQL visual browser [35] and an example to query the Knowledge Base and get RDF triples.

**Figure 6.**
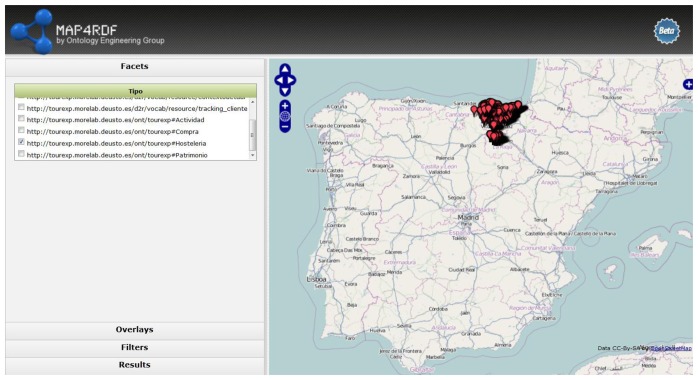
Example of map4rdf application querying TourExp SPARQL endpoint.

Figure 6 shows an example of application called map4rdf deployed in TourExp [36] that uses the TourExp SPARQL endpoint to discover all the entities from TourExp data model with coordinates and locate their entries in a map.

#### 4.2.5. Knowledge Inference Module

This component is in charge of obtaining and deducing new information from the context data stored by the Gathering module. The inferred information can be used by context information consumers like the recommendation system for suggesting accurate tourist activities or resources to a costumer/visitor depending on his/her context, or even the content manager, as an editing help tool for tourist resources providers in the process of associating their products to context parameters or travel profiles.

Some batch scripts are designed for implementing this inferring functionality, including tasks like:

- filling gaps in customers' profiles from their Facebook profiles,
- detecting new relations among customers' profiles using their Facebook contacts or other context data like preferences, restrictions, items selected as favourites, bookings, etcetera,
- detecting new relations between customers' profiles and traveler profiles (defined by range of ages, gender, country, educational background, *etc.*),
- detecting new relations between traveler profiles and types of travel profiles (defined by budget, travel motivation, travel mates, *etc.*),
- detecting new relations between types of travel profiles and types of experiences, activities, packages of activities and tourist resources (accommodations, restaurants, cultural heritage and shopping areas).

Since the TourExp system is not finished yet, there are no real data to work with so it is impossible to determine real travel and traveller profiles. However, some travel and traveller profiles have been defined using statistical data from some public reports of the Basque Tourism Agency.

## 5. Conclusions

This research work presents a new approach to parameterize, model and share context data related to a visitor or tourist on the move, as well as the architecture for a context information management platform based on this context data model. Human, environmental and system factors are the main domains that have been taken into account in order to represent the context information about a visitor at a specific location and time. Also, the bookings of the visitor are being tracked in order to create a better recommendation system based on context data. All the entities that represent context data have been modeled on a relational database that has been mapped to different vocabularies. This context data can be query by other modules of the TourExp system and also by third party services by means of RESTful web services that returns JSON or XML files or even using a SPARQL endpoint that returns RDF triples. Besides, the SPARQL endpoint is also a powerful tool that allows complex queries to get implicit knowledge that is not made explicit using a traditional relational database. This functionality can be seen also as a way to publish the content of the TourExp database as Linked Open Data. These context data are processed by the knowledge inference module to classify customers in different travelers' profiles. Besides, they are also exploited by the TourExp recommendation system in order to perform better suggestions to the users, which can be done using all or part of the human, environmental and/or system factors defined in the TourExp context data model and depending on the filters selected on the mobile application. Basque Tourism Agency is interested in validating these developments with some tourist providers located in a basque region called Gorbeialde.

## Figures and Tables

**Table 1.** List of vocabularies linked from TourExp Data Model.

**Prefix**	**URI**
tourexp-owl	http://tourexp.morelab.deusto.es/ont/tourexp#
contology-owl	http://tourexp.morelab.deusto.es/ont/contology#
wgs84_pos	http://www.w3.org/2003/01/geo/wgs84_pos#
dbpedia-owl	http://dbpedia.org/ontology/
dbpedia-prop	http://dbpedia.org/property/
dbpedia	http://dbpedia.org/resource/
vocab	http://tourexp.morelab.deusto.es/d2r/vocab/resource/

**Table 2.** Example of mappings for the “Accommodation” entity and columns (tourist resource).

**Entity**	**Column**	**Prefix:Term**
Accommodation		tourexp-owl:Accommodation
	mainImage	dbpedia-prop:image
	localityCode	contology-owl:isLocatedAt
	phoneNumber	dbpedia-prop:phone
	postalCode	dbpedia-prop:postcode
	fax	dbpedia-prop:fax
	phoneNumber2	dbpedia-prop:phone
	name	dbpedia-owl:Name
	category	dbpedia-owl:starRating
	provinceCode	dbpedia-owl:provinceid
	mail	dbpedia-prop:mail
	contactPerson	dbpedia-prop:contact
	url	dbpedia-prop:url
	coordinates	dbpedia-prop:coordinates
	numOfRooms	dbpedia-owl:numberOfRooms
	numOfSuites	dbpedia-owl:numberOfSuites
	openingDate	dbpedia-owl:openingDate
	latitude	wgs84_pos:latitude
	longitude	wgs84_pos:long
	localityID	contology-owl:isLocatedAt

**Table 3.** Example of mappings for the “CurrentContext” entity and columns (context information).

**Entity**	**Column**	**Prefix:Term**
CurrentContext		vocab:CurrentContext
	travelMotivation	contology-owl:Motivation
	typeOfDevice	contology-owl:Device
	numOfNights	contology-owl:TemporalUnit
	currentLatitude	wgs84_pos:lat
	currentLongitude	wgs84_pos:long
	customerID	contology-owl:Visitor

**Table 4.** RESTful web services for storing context data.

**Gathering Service**	**URL for PUT Requests**	**Required Parameters**
Customer's location	[URLBase]/PerpetuaDatos/GeoData/{idUser}: user's identifier	longitude
		latitude

Current weather conditions	[URLBase]/weather/{idUser}: user's identifier	longitude
		latitude

Customer's profile	[URLBase]/PerpetuaDatos/UserProfile/{idUser}: user's identifier	name
		lastName
		phoneNumber
		gender
		dateOfBirth
		language
		educationBackground
		occupation
		countryCode
		provinceCode

Customer's travel profile	[URLBase]/PerpetuaDatos/TripProfile/{idUser}: user's identifier	budget
		ttravelMotivation
		typeOftravel
		typeOfDevice
		gps
		typeOfAccommodation
		numOfNights

Customer's Facebook profile	[URLBase]/PerpetuaDatos/FacebookProfile/{id}: user's Facebook ID	customerID

Customer's favourite items	[URLBase]/PerpetuaDatos/Favourite/{idUser}: user's identifier	typeOfFavouriteItem
		itemID
		score

**Table 5.** RESTful web services of the Query module.

**Query Functionality**	**Required Parameters id: Customer's Identifier**
Search	/search
Profile	/{id}
Restrictions	/{id}/restricciones
Environmental preferences	/{id}/entorno
Facebook profile	/{id}/facebook
Check-ins	/{id}/checkins
Facebook contacts	/{id}/contactos
Friends list	/searchAmigos?id={id}
Current context	/{id}/contexto
Historical context data	/{id}/tracking
Historical data about activities	/{id}/trackingA
Historical data about packages	/{id}/trackingP
Booked activities	/{id}/actividadesR
Booked packages	/{id}/paquetesR
Favourite accommodations	/{id}/alojamientosF
Favourite restaurants	/{id}/restaurantesF
Favourite shopping areas	/{id}/comprasF
Favourite cultural heritage	/{id}/patrimonioF
Favourite activities	/{id}/actividadesF
Favourite packages	/{id}/paquetesF
Favourite type of experiences	/{id}/experienciasF
